# Differential DNA Methylation Landscape in Skin Fibroblasts from African Americans with Systemic Sclerosis

**DOI:** 10.3390/genes12020129

**Published:** 2021-01-20

**Authors:** DeAnna Baker Frost, Willian da Silveira, E. Starr Hazard, Ilia Atanelishvili, Robert C. Wilson, Jonathan Flume, Kayleigh L. Day, James C. Oates, Galina S. Bogatkevich, Carol Feghali-Bostwick, Gary Hardiman, Paula S. Ramos

**Affiliations:** 1 Department of Medicine, Division of Rheumatology and Immunology, Medical University of South Carolina, Charleston, SC 29425, USA; bakerde@musc.edu (D.B.F.); atanelis@musc.edu (I.A.); jof63@musc.edu (J.F.); oatesjc@musc.edu (J.C.O.); bogatkev@musc.edu (G.S.B.); feghalib@musc.edu (C.F.-B.); 2 Institute for Global Food Security, School of Biological Sciences, Queens University Belfast, Belfast BT9 5DL, UK; w.dasilveira@qub.ac.uk (W.d.S.); g.hardiman@qub.ac.uk (G.H.); 3 Computational Biology Resource Center, Medical University of South Carolina, Charleston, SC 29425, USA; hazardes3@gmail.com; 4 Department of Pathology and Laboratory Medicine, Medical University of South Carolina, Charleston, SC 29425, USA; relaxingbob@gmail.com; 5 Stevenson University, Stevenson, MD 21117, USA; kayleighday25@gmail.com; 6 Rheumatology Section, Ralph H. Johnson VA Medical Center, Charleston, SC 29425, USA; 7 Department of Public Health Sciences, Medical University of South Carolina, Charleston, SC 29425, USA

**Keywords:** systemic sclerosis, African American, DNA methylation, genome, skin fibroblasts

## Abstract

The etiology and reasons underlying the ethnic disparities in systemic sclerosis (SSc) remain unknown. African Americans are disproportionally affected by SSc and yet are underrepresented in research. The aim of this study was to comprehensively investigate the association of DNA methylation levels with SSc in dermal fibroblasts from patients of African ancestry. Reduced representation bisulfite sequencing (RRBS) was performed on primary dermal fibroblasts from 15 SSc patients and 15 controls of African ancestry, and over 3.8 million CpG sites were tested for differential methylation patterns between cases and controls. The dermal fibroblasts from African American patients exhibited widespread reduced DNA methylation. Differentially methylated CpG sites were most enriched in introns and intergenic regions while depleted in 5′ UTR, promoters, and CpG islands. Seventeen genes and eleven promoters showed significant differential methylation, mostly in non-coding RNA genes and pseudogenes. Gene set enrichment analysis (GSEA) and gene ontology (GO) analyses revealed an enrichment of pathways related to interferon signaling and mesenchymal differentiation. The hypomethylation of *DLX5* and *TMEM140* was accompanied by these genes’ overexpression in patients but underexpression for lncRNA *MGC12916*. These data show that differential methylation occurs in dermal fibroblasts from African American patients with SSc and identifies novel coding and non-coding genes.

## 1. Introduction

Systemic sclerosis (SSc or scleroderma) is a rare, multisystem, connective tissue disease characterized by cutaneous and visceral fibrosis, immune dysregulation, and vasculopathy. Patients are commonly classified into two main subsets, limited cutaneous SSc (lcSSc) and diffuse cutaneous SSc (dcSSc), with dcSSc having a worse prognosis [1]. Relative to individuals of European ancestry, individuals of African ancestry are more likely to develop SSc, to be diagnosed with dcSSc, and to experience higher disease severity, greater morbidity, reduced survival, and earlier death [2,3,4,5,6,7,8]. This higher disease burden in African Americans is not fully explained by differences in socioeconomic status or access to health care [8,9].

The etiology of SSc and the factors underlying its ethnic disparities remain elusive. Genetic and epigenetic studies conducted mostly in individuals of European ancestry uncovered multiple loci associated with SS [10]. A role of DNA methylation in SSc is supported by a X chromosome gene methylation analysis of peripheral blood mononuclear cells [11]; the quantification of global methylation in whole blood [12]; as well as genome-wide DNA methylation analyses of dermal fibroblasts [13], whole blood [14], and CD4+ T cells [15,16]. Different ancestral populations exhibit DNA methylation differences [17,18,19,20,21,22,23,24] that are partially explained by their distinct genetic ancestry, thus environmental factors not captured by genetic ancestry are significant contributors to the variation in methylation [19].

In order to understand the pathogenesis of SSc in patients of African ancestry, we assessed the DNA methylation profiles of dermal fibroblasts from African American patients and controls by RRBS, which has a high sensitivity and specificity to detect changes in DNA methylation in genes, promoters, CpG islands, and repetitive regions [25,26]. We then integrated the data with the gene expression of the top differentially methylated genes from the same subjects. This study is the first to unveil the genome-wide patterns of differential methylation in skin fibroblasts from African American patients with SSc.

## 2. Materials and Methods

### 2.1. Subjects

A total of 15 SSc cases and 15 healthy controls were recruited for this study. All the participants were self-reported African American and the patients met the 2013 ACR/EULAR classification criteria for SSc. The cases and controls were age-balanced within 5 years. This study was approved by the Institutional Review Board at the Medical University of South Carolina on 9 April 2014 (application number Pro# 33636). Informed consent was obtained from all the participants. All the research included in this manuscript conforms with the Declaration of Helsinki.

### 2.2. Primary Dermal Fibroblast Isolation and Culture

Primary dermal fibroblasts were isolated from 3 mm skin biopsies obtained from the involved forearm skin and cultured as described [27]. Cells were cultured for 3 passages, then DNA and RNA were isolated using the DNeasy and RNeasy kits (Qiagen, Germantown, MD, USA) following the manufacturer’s protocols.

### 2.3. Reduced Representation Bisulfite Sequencing (RRBS)

RRBS was performed using the Ovation^®^ RRBS Methyl-Seq System 1–16 (NuGEN Technologies, Inc., San Carlos, CA, USA), following the manufacturer’s procedure. We generated DNA methylation data for over 5 million CpGs in each sample and between 10× to 40× coverage in CpG sites.

### 2.4. Genome-Wide DNA Methylation Data Analysis

Alignment and methylation calling were performed using Bismarck v0.16.3 and the GRCh37/hg19 reference genome [28]. Data were filtered, normalized, and analyzed with RnBeads v1.6.1 [29]. A differential methylation analysis was conducted at the CpG, promoter, and gene levels (including RNA, pseudo-, and protein-coding genes) [29]. Genes and promoters were defined by Ensembl (ensembl.org), and CpG islands were defined as the CpG island track of the UCSC Genome Browser (genome.ucsc.edu). As implemented in RnBeads, CpG site *p*-values were computed using the linear models in the limma package. For gene regions, promoter regions, and CpG islands, the mean of the mean methylation levels for cases and controls across all sites in a region was computed, as well as the following three quantities: the mean difference in means (MDM) across all sites in a region, the mean of quotients in mean methylation across all sites in a region, and a combined *p*-value calculated from all site *p*-values in the region [29]. Each gene, promoter, and CpG island was assigned a rank based on these three criteria. A combined rank is computed as the maximum (i.e., worst) value among the three ranks. The smaller the combined rank for a region, the more evidence for differential methylation it exhibits. All genes, promoters, and CpG islands were ranked based on this Combined Score approach implemented in RnBeads [29]. Of note, while a negative MDM represents hypomethylation in cases relative to controls, a positive MDM denotes hypermethylation in cases relative to controls.

### 2.5. Genomic Annotation Enrichment Analysis

To annotate the position of each CpG to the corresponding genomic location, we used the *annotatePeaks.pl* program of HOMER v4.9.1 with annotation from the hg19 human genome assembly [30]. CpGs were annotated to promoter, transcription termination site (TTS), exon, intron, 5′ UTR exon, 3′ UTR exon, intergenic, CpG island, repeat elements, and other detailed annotations using default region definitions. HOMER uses annotations based on the UCSC Genome Browser (genome.ucsc.edu). To investigate the distribution of differentially methylated CpGs (DMC) in different genomic locations, all CpGs that met an FDR-adjusted *p*-value < 0.4 were used to compare their localization in different genomic locations as provided by HOMER’s annotations [30]. Odds ratios (ORs), 95% confidence intervals (CI), and *p*-values were computed against the general distribution of the 3,870,251 CpGs of our dataset using GraphPad Prism v9.

### 2.6. Gene Set Enrichment Analysis (GSEA)

A gene set enrichment analysis GSEA was performed to determine whether a priori defined sets of genes (e.g., pathways) are significantly enriched in the list of genes ranked by their correlation with the disease. The full ranked lists of genes and promoters generated by RnBeads’ Combined Score approach [29] were used as inputs to GSEA Desktop v3.0 [31,32]. The genes were ranked by their differential methylation between cases and controls (hyper- and hypomethylated), and the Reactome Pathway Knowledgebase (reactome.org) [33] was used as the gene set. An enrichment score statistic represents the enrichment of Reactome pathways in genes that are hyper- or hypomethylated in patients, and the significance of the pathway enrichment score is estimated by an empirical phenotype-based permutation test procedure [31,32]. The threshold for statistical significance was defined as FDR ≤ 0.25, as recommended [31,32].

### 2.7. Gene Ontology (GO) Enrichment Analysis

Enrichment analysis for GO terms associated with the top-ranking differentially methylated genes and promoters was performed using RnBeads v1.6.1 [29]. A GO enrichment analysis of biological process (BP) was conducted separately on each of the 100 hypo- and hypermethylated genes and promoters. The enrichment of GO BP terms associated with the top ranking genes and promoters was determined by a hypergeometric test implemented in RnBeads [29]. 

### 2.8. Gene Expression Analysis

Using the available cultured fibroblast samples from the same 15 SSc cases and 14 healthy controls, cDNA was prepared using the Superscript IV First Strand synthesis system (ThermoFisher, Waltham, MA, USA) from 1 μg of isolated RNA. qPCR was performed using the Taqman Real-Time PCR master mix (Applied Biosystems, Foster City, CA, USA). All the samples were run in duplicate using an Applied Biosystems Real-Time PCR System and analyzed using the StepOne Plus Applied Biosystems software. The gene quantification cycle values were normalized to β 2 microglobulin (B2M) expression using the ΔΔCT method to obtain relative cell equivalents. All the primers were purchased from ThermoFisher Scientific (Waltham, MA, USA): *CDA* (Hs00156401_m1); *TMEM140* (Hs00251020_m1); *ACKR4* (Hs00664347_s1); *DLX5* (Hs01573641_m1); *FAM180B* (Hs03988397_m1); *MGC12916* (Hs04419380_s1); *LOC102724927* (Hs04395955_s1); *LOC101929882* (Hs04938653_m1); *B2M* (Hs00187842_m1). Statistical significance was determined using the Mann–Whitney test and defined as *p*-values ≤ 0.05.

## 3. Results

### 3.1. Subject Characteristics

The clinical and demographic characteristics of the volunteer African ancestry SSc patients and healthy controls are summarized in Table 1. Most patients were female with relatively early disease (mean duration of 5 years). Most presented with dcSSc, having more extensive skin disease involving the proximal limbs and trunk and no concomitant rheumatic disease. One patient had the rarer SSc sine scleroderma (ssSSc), which is the total or partial absence of cutaneous manifestations but the presence of internal organ involvement and/or serologic findings consistent with SSc.

### 3.2. Differentially Methylated Sites and Genes

**Figure 1:**
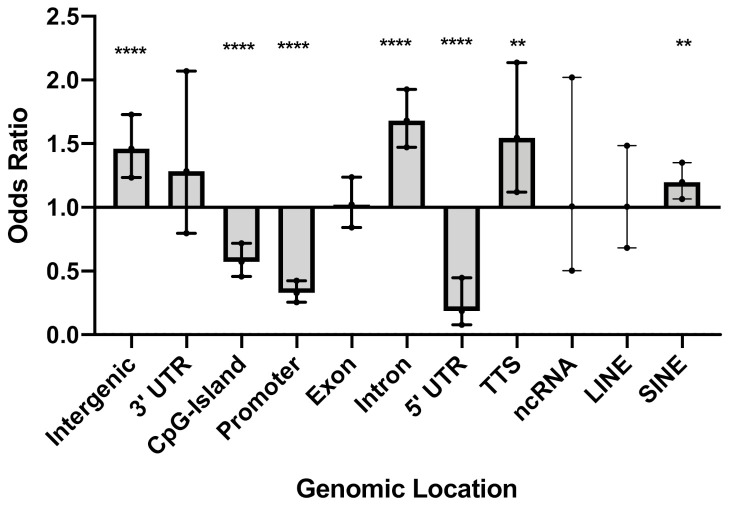
Genomic location of differentially methylated CpGs (DMC) that met an adjusted *p*-value < 0.4. Odds ratio (OR), 95% confidence intervals (CI), and *p*-values were computed against the general distribution of the 3,870,251 CpGs of our dataset using GraphPad Prism v9. Error bars represent the 95% CI. OR indicates the enrichment or depletion of DMCs in each region. Transcription termination site (TTS); non-coding RNA (ncRNA); long interspersed nuclear elements (LINE); short interspersed nuclear elements (SINE). ** *p* ≤ 0.01, **** *p* ≤ 0.0001.

Over 3.8 million CpG sites were tested for differential methylation between SSc cases and controls (Supplementary Figure S1). A total of 1180 differentially methylated CpGs (DMCs), which corresponds to 0.03% of all cytosines tested, meet an FDR-adjusted *p*-value < 0.4. The rationale for the FDR setting was guided by the desire to perform a system-level analysis and include as many CpGs sites as possible, as well as our previous studies demonstrating that this threshold permits a sensitive analysis at a system level of genes that are relevant to the underlying biology of the trait [34,35]. Patients exhibited widespread hypomethylation throughout the genome, with over 85% of CpGs that met an FDR-adjusted *p*-value < 0.4 showing decreased methylation in the cultured skin fibroblasts from the patients compared to the controls. We first sought to investigate any potential enrichment (or conversely, underrepresentation) of DMCs in defined genomic regions. Among the 1180 DMCs that met an adjusted *p*-value < 0.4, there was an overrepresentation of DMCs in introns (OR = 1.7, *p *< 0.0001), intergenic regions (OR = 1.5, *p* < 0.0001), transcription termination sites (TTS) (OR = 1.5, *p* = 0.007), and short interspersed nuclear elements (SINE) (OR = 1.2, *p* = 0.003) (Figure 1 and Supplementary Table S1). Notably, there was a depletion of DMCs in 5′ UTR (OR = 0.2, *p* < 0.0001), promoters (OR = 0.3, *p* < 0.0001), and CpG islands (OR = 0.6, *p* < 0.0001) (Figure 1 and Supplementary Table S1). In most of the genomic regions, the majority of DMCs were hypomethylated in the patients compared to controls. In contrast with other genomic regions, in CpG islands 71% of the DMCs were more methylated in patients than the controls.

The Combined Score approach implemented in RnBeads v1.6.1 [29] was used to independently identify differentially methylated genes, promoters, and CpG islands. Of note, RnBeads uses the definitions of genes and promoters from Ensembl and CpG islands from the UCSC Genome Browser. A total of 197 (out of 30,771) genes, 112 (out of 29,720) promoters, and 97 (out of 24,117) CpG islands were identified and ranked using this approach. The gene and promoter regions identified are shown in Table 2. A total of 9 CpG islands, 17 genes (including RNA, pseudo- and protein-coding genes), and 11 promoters showed significant differential methylation levels between cases and controls at the gene level. The top differentially methylated genes constitute mostly non-coding RNA genes (42%), followed by pseudogenes (27%) and then protein-coding genes (19%) (Table 2). Among the protein-coding genes, *cytidine deaminase (CDA)*, a marker of monocyte/macrophage differentiation [36], is involved in innate immunity pathways. *Atypical chemokine receptor 4 (ACKR4)* is involved in chemokine signaling [37]. *Distal-less homeobox 5 (DLX5)* is a transcription factor involved in bone development and the morphogenesis of connective tissue [38]. The functions of the remaining genes are currently unknown.

### 3.3. Gene Set Enrichment Analysis (GSEA)

To gain insight into the most differentially methylated genes and promoters, GSEA [31,32] was conducted to predict biologically relevant Reactome pathways [33]. Table 3 lists all Reactome pathways with an FDR ≤ 0.25, as recommended by GSEA [32]. This analysis highlighted an immune pathway (immunoregulatory interactions between a lymphoid and a non-lymphoid cell) to be overrepresented in the set of hypermethylated genes, while metabolism pathways (glucuronidation, chondroitin sulfate dermatan sulfate metabolism) showed enrichment among hypomethylated genes. Pathways involved in cell development (the regulation of β cell development and gene expression) and cell signaling (gap junction trafficking, the activation of kainate receptors upon glutamate binding, G β:γ signaling), were also enriched among hypomethylated genes.

### 3.4. Gene Ontology (GO) Enrichment Analysis

To further aid in the interpretation of the differentially methylated genes and promoters, we performed an enrichment analysis for GO terms associated with the top-ranking genes and promoters in Table 4. Multiple development and morphogenesis, immune, and metabolic-related terms show enrichment. Meanwhile, hypomethylated genes are enriched for GO terms associated with interferon (IFN) signaling (type I IFN signaling pathway, *p* = 8.0 × 10^−4^; response to type I IFN, *p* = 8.0 × 10^−4^) and hypermethylated genes are enriched for GO terms associated with mesenchyme and epithelial development and cell differentiation (epithelial to mesenchymal transition, *p* = 1.0 × 10^−4^; nephron tubule formation, *p* = 1.0 × 10^−4^; mesenchymal cell differentiation *p* = 1.0 × 10^−4^) (Table 4).

### 3.5. Comparison of DNA Methylation with Previous Reports in Dermal Tissues

The 28 genes and promoters identified in this study (Table 2) were compared to results from published genome-wide DNA methylation [13] and gene expression studies [39,40,41,42,43,44,45,46,47,48,49] in cultured dermal fibroblasts or skin biopsies. Of our top genes and promoters, two CpGs in *distal-less homeobox 5 (DLX5)* were reported as hypermethylated in skin fibroblasts from dcSSc patients [13], which is consistent with our results.

When compared to gene expression profiling studies in cultured dermal fibroblasts or skin biopsies, *DLX5* was reported to be under-expressed in patients with SSc [43], while *transmembrane protein 140* (*TMEM140*) was reported to be overexpressed in patients with SSc [43] and correlated with the modified Rodnan skin thickness score (mRSS) in dcSSc patients [47].

When compared to the genes with compelling evidence of genetic association with SSc [10], none of our top 28 genes has been previously reported. Of note, these genome-wide DNA methylation [13] and gene expression studies [39,40,41,42,43,44,45,46,47,48,49] in skin-related tissues, as well as genetic association studies [10], were all performed in individuals of mostly European ancestry.

### 3.6. Gene Expression of Differentially Methylated Genes

**Figure 2:**
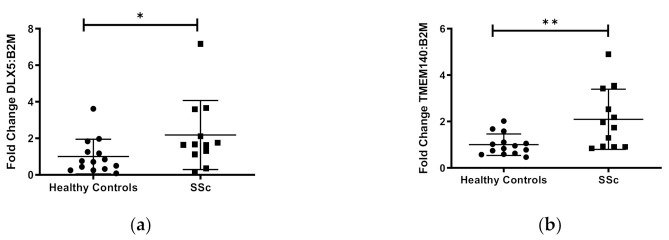

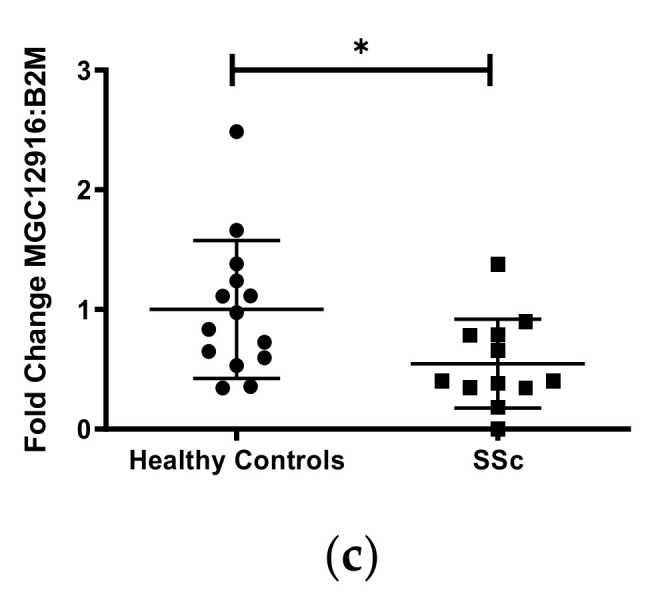
Transcript levels of three differentially expressed genes in cultured skin fibroblasts. Among eight genes that were chosen for analysis, three genes demonstrated significantly differentiated expression in African ancestry SSc patients compared to controls: (**a**) DLX5, (**b**) TMEM140, and (**c**) MGC12916. Participant classification is detailed on the *x*-axis, while gene transcript-level fold change is expressed on the *y*-axis. Figure is representative of 14 healthy controls and 15 SSc patients. * *p* ≤ 0.05, ** *p* ≤ 0.01.

To evaluate the functional effects of DNA methylation on gene expression in our sample of African American subjects, we performed qPCR on the five protein-coding genes (*CDA, TMEM140, ACKR4, DLX5, FAM180B*) and three long non-coding (lnc) RNA genes (*MGC12916, LOC102724927, LOC101929882*). These genes were chosen based on their known functions, an increased number of CpG sites detected (>30), and/or detectable transcripts from primary dermal fibroblasts using the RNA isolation/purification technique outlined in the methods section. Of the eight gene transcripts quantified, *DLX5, TMEM140,* and *MCG12916*, showed significant differential expression in cases compared to controls (Figure 2). Although these three genes showed hypermethylation, both the *DLX5* and *TMEM140* steady-state transcript levels were increased, while the *MCG12916* steady-state transcript levels were decreased in patients compared to controls (Figure 2a–c).

## 4. Discussion

This is the first study investigating patterns of differential methylation in primary skin fibroblasts from African American patients with SSc. We found widespread reduced DNA methylation in patients compared with healthy controls, consistent with what has been previously reported in skin fibroblasts from SSc patients of mostly European ancestry [13], and peripheral blood from Black South African patients with SSc [12].

Our findings show novel, top differentially methylated genes constituting mostly non-coding RNA genes and pseudogenes, with the function of most genes currently unknown. Only three protein-coding genes were amongst the top results: *CDA* and *ACKR4* with known roles in immune pathways, and *DLX5* with roles in cell development and proliferation. *DLX5* was previously reported as hypermethylated in skin fibroblasts from dcSSc patients [13], which is consistent with our results. However, the previous study analyzed DNA methylation using the HumanMethylation450K array. Our study is based on RRBS, which tested eight times more CpGs than those present on the HumanMethylation450K array used in the previous genome-wide study of skin fibroblasts [13]. Thus, these methods are not directly comparable. Because the array contains only 2% of the CpGs we tested [50], minimal overlap can be expected. In addition, extensive differences in DNA methylation are known to exist between individuals of African and European ancestry [17,18,19,20,21,22,23,24], due to both variation in genetic ancestry and environmental factors [19], with Africans showing a higher DNA methylation than Europeans [20]. These differences help explain the new findings and minimal overlap with previous reports.

*DLX5, TMEM140,* and *MCG12916* exhibited concomitant differential gene expression in the same primary dermal fibroblasts among the differentially methylated genes. While these genes exhibited hypermethylation, *DLX5* and *TMEM140* showed overexpression, while *MCG12916* showed downregulation in the same individuals. This is not surprising, as the correlation between DNA methylation and gene expression is positive or negative and is tissue or context specific, in that the local DNA sequence and genomic features largely account for local patterns of methylation [51,52,53]. There is great variation in the quantitative impact of DNA methylation on gene expression among different cell types, with both positive and negative correlations between expression levels and CpG methylation levels [20,54,55,56,57]. Thus, the variable correlation between methylation patterns and gene expression is well established. Our results show that the hypomethylation of CpGs was prominent in all regions but CpGs islands, where DMCs were hypermethylated. DMC sites were enriched in introns, intergenic regions, TTS, and SINE, while depleted in 5′ UTR, promoters, and CpG islands. The overrepresentation of DMCs in introns has been previously reported in whole blood and neutrophils from systemic lupus erythematosus (SLE) patients [58,59], and in CD4+ T cells from SSc patients [15], while their overrepresentation in intergenic regions has also been found in CD4+ T cells from SSc patients [16]. Similarly, the underrepresentation of DMCs in promoters, CpG islands, and 5′ UTR has been previously reported in whole blood and neutrophils from SLE patients [58,59], and that of CpG islands is also found in CD4+ T cells from SSc patients [16]. Because CpG sites preferentially located in enhancers are reported to mediate gene expression, not in the promoters, this further supports a modest role of promoters in epigenetic regulatory mechanisms [20].

Interestingly, despite the differences in tissue and patient characteristics, *TMEM140* was reported as overexpressed in skin biopsy specimens from patients with SSc [43] and correlated with the mRSS in dcSSc patients [60], which corroborates our findings. *TMEM140* was identified as an IFN-inducible gene in cells infected with the human T-lymphocytic virus [61]. Since IFN signaling occurs in SSc [62,63,64,65] and is confirmed in this report, the overexpression of *TMEM140* in AA SSc dermal fibroblasts may be in response to the IFN signature observed in SSc.

On the other hand, *DLX5* was reported as under-expressed in skin biopsy specimens from patients with SSc [43]. The different outcomes of gene expression for *DLX5* between the experiments could be the result of measuring gene expression in one cell type vs. across multiple cell types in skin biopsies, as well as underlying ancestral differences in gene expression. The inhibition of DLX5 in a uremic model of renal fibrosis causes a decreased expression of Notch receptors, ligands, and target genes [66]. Because Notch signaling is active in skin fibroblasts isolated from SSc patients and contributes to fibrosis in animal models [67,68], DLX5 may also regulate fibrosis through Notch signaling in SSc.

Although multiple lncRNAs have been reported as dysregulated in SSc patient tissues [69], to our knowledge this is the first report that *MGC12916* has differential gene methylation and expression in primary dermal fibroblasts from African American patients with SSc.

To elucidate the underlying biological processes associated with SSc, GSEA and GO enrichment analyses were conducted. Among the hypomethylated regions, both GSEA and GO enrichment analyses showed the enrichment of immune pathways, with the GO analysis showing an enrichment in type I IFN signaling. Patients with SSc have excessive IFN and an IFN signature that correlates to early and more severe disease [62,63,64,65]. IFN is also pathogenic in SSc, since exogenous exposure to IFNα or IFNβ leads to its development [70,71,72,73]. The IFN regulatory factor 7 promoter (IRF7) is hypomethylated in SSc peripheral blood mononuclear cells [74], supporting the link of IFN signaling and gene hypomethylation in SSc.

Among hypermethylated regions, GSEA showed an enrichment of metabolism, cell development, and cell signaling pathways, and a GO enrichment analysis revealed an enrichment in specific pathways related to mesenchyme and epithelial development and cell differentiation. The top enriched GO term among hypermethylated regions, endothelial-mesenchymal cell transition (EMT), is consistent with the current hypothesis that EMT likely influences SSc disease characteristics, including endothelial cell dysfunction, dermal fibrosis, and interstitial lung disease [75,76]. To our knowledge, this is the first reported association between gene and promoter hypermethylation and mesenchymal cell differentiation in SSc. Thus, our GESA and GO analyses correlate with previous data regarding known pathways in SSc.

There are limitations to this study. First, although we are the first to analyze patterns of DNA methylation in dermal fibroblasts in African Americans, the sample size is modest. Nevertheless, with 15 SSc patients it is comparable to previous genome-wide DNA methylation analyses focused on skin fibroblasts (*n* = 12 SSc patients) [13], whole blood (*n* = 27 SSc patients) [14], and CD4+ T cells (*n* = 9 patients) [15], which included primarily individuals of European ancestry. Second, SSc is a rare disease with a prevalence of only 49,000 US adults [77], and there is currently no existing cohort or repository of samples from African American patients that can be leveraged to replicate and validate our results. Future studies expanded to multiple centers are needed. Third, the comparison of our results to those previously reported in European ancestry patients is hindered by differences in the analytic methods. Future studies including diverse individuals with measures of genetic ancestry as well as self-reported race, ethnicity, and other social and environmental exposures will ensure the validity and relevance of these findings for patients of all backgrounds. Fourth, and inherent to all epigenomic studies, we cannot exclude the possibility of reverse causation, or whether the DNA methylation changes are a cause or an effect of SSc. Future longitudinal studies, as well as studies comparing skin fibroblasts between affected and unaffected skin in patients and between affected and unaffected relatives will help to elucidate the role of DNA methylation in disease etiology. Fifth, it is possible that the DNA methylation changes are due to genetic variation. We lack genotypic data on these samples, but note that none of the top differentially methylated genes has been previously reported to be associated with SSc. We recognize that it is difficult to account for all lifestyle factors that could affect DNA methylation (i.e., diet, physical activity, body weight, smoking, medications, etc.) [78]. Our samples were balanced relative to smoking and age, so their confounding effects are minimized. Finally, we do not know the role of *DLX5, TMEM140,* and *MCG12916* in SSc, but future gene silencing studies to inhibit the expression of these genes in primary dermal SSc fibroblasts will help elucidate their function in this cell type. In spite of these limitations, these findings identify novel loci in SSc and highlight candidate genes for further research.

## 5. Conclusions

This first genome-wide DNA methylation study of skin fibroblasts from SSc patients of African ancestry identified novel differentially methylated sites and genes. These include sites with evidence of altered methylation in protein-coding, lncRNA, and pseudogenes and concomitant differential expression in *MGC12916, DLX5*, and *TMEM140.* Although this cross-sectional study cannot separate causality from response to disease, it identifies DNA methylation alterations in genes and pathways that are important in SSc, showing that distinct DNA methylation changes underlie SSc in African Americans. These findings provide a foundation for further research to determine the functional consequences of the differentially methylated loci. Given the reversible nature of epigenetic marks, these loci might represent attractive targets for the treatment or prevention of autoimmune- and/or fibrotic-related diseases.

## Figures and Tables

**Table 1:** Demographic and clinical characteristics of the study participants.

	Patients (*n* = 15)	Controls (*n* = 15)
Age at enrollment (mean ± SD)	44.4 ± 9.7	45.6 ± 9.9
Female, *n* (%)	10 (67%)	12 (80%)
dcSSc, *n* (%)	14 (93%)	NA
ssSSc, *n* (%)	1 (7%)	NA
Raynaud’s Phenomemon, *n* (%)	15 (100%)	NA
Disease duration (mean ± SD)	5.3 ± 5.2	NA
mRSS (mean ± SD) ^1^	18.6 ± 9.1	NA
ILD, *n* (%)	3 (20%)	NA
PH/PAH, *n* (%)	6 (40%)	NA
Overlap SLE, *n* (%)	2 (13%)	NA
Anti-topoisomerase I, *n* (%) ^2^	5 (46%)	NA
Anti-RNA polymerase III, *n* (%) ^3^	1 (11%)	NA
Immunosuppressive medications, *n* (%)	10 (67%)	NA
Smoker at enrollment, *n* (%) ^4^	1 (7%)	1 (7%)

SSc: systemic sclerosis; dcSSc: diffuse cutaneous SSc; ssSSc: sine SSc; mRSS: modified rodnan skin score; ILD: interstitial lung disease; PH/PAH: pulmonary hypertension/pulmonary arterial hypertension; SLE: systemic lupus erythematosus. ^1^: Assessed for all patients with dcSSc at enrollment or within 3 months (for 3 patients); ^2^: Measured for 11 (73%) of patients at enrollment or within 1 year (1 patient); ^3^: Measured for 9 (60%) of patients at enrollment or within 1 year (3 patients); ^4^: Disclosed for all patients and 13 (87%) controls.

**Table 2:** Gene and promoter regions below the Combined Score cutoff.

Symbol	Gene Type	Chr	Position (kb)	MDM	*n* Sites	Rank
**Genes**						
RPL30P7	Pseudogene	5	10,489–10,489	0.31	1	25
MGC12916	RNA gene (lncRNA)	17	14,207–14,209	0.23	31	60
LINC01227	RNA gene (ncRNA)	16	80,601–80,607	−0.21	1	80
ENSG00000255342	Uncategorized (lncRNA)	11	123,007–123,007	0.22	11	81
ENSG00000227930	RNA gene	7	23,931–23,937	−0.2	2	84
ENSG00000230104	Uncategorized (lncRNA)	2	173,539–173,540	0.22	1	131
LOC102724927	RNA gene (ncRNA)	16	3998–4000	−0.14	5	132
ENSG00000229472	-	20	32,669–32,670	0.18	2	133
MIR5587	RNA gene (miRNA)	16	585–585	0.22	2	141
LOC105379365	RNA gene (ncRNA)	8	34,032–34,042	0.17	2	146
LOC402634	Pseudogene	7	2433–2434	0.17	2	163
NEK2P4	Pseudogene	2	131,935–131,937	0.2	7	173
NCRNA00250	RNA gene (ncRNA)	8	135,850–135,855	−0.19	1	180
DLX5	Protein coding	7	96,650–96,654	0.17	114	192
LOC101929882	RNA gene (ncRNA)	2	10,179–10,181	0.13	2	194
FAM180B	Protein coding	11	47,608–47,611	0.17	6	195
LOC100652792	Pseudogene	15	93,306–93,307	0.16	4	197
**Promoters**						
CDA	Protein coding	1	20,914–20,916	−0.26	1	7
TAF5LP1	Pseudogene	17	33,824–33,826	−0.22	8	23
LINC00619	RNA gene (ncRNA)	10	44,339–44,341	−0.25	1	29
RPL30P7	Pseudogene	5	10,487–10,489	−0.31	1	45
SNORA25	RNA gene (snoRNA)	13	106,549–106,551	−0.23	3	72
ENSG00000241456	RNA gene	7	151,123–151,125	0.17	3	74
ENSG00000229974	-	7	134,832–134,834	0.18	2	94
TMEM140	Protein coding	7	134,831–134,833	0.18	2	94
LOC100420018	Pseudogene	11	35,990–35,992	0.37	1	101
ACKR4	Protein coding	3	132,315–132,317	−0.23	1	110
ENSG00000255342	Uncategorized (lncRNA)	11	123,007–123,009	−0.22	11	112

Genes and promoters are ranked based on the RnBeads’ Combined Score approach [29]. MDM is the mean difference in mean methylation levels across all sites in a region, and *n* sites is the number of sites associated with the region. A negative MDM represents hypomethylation in cases relative to controls, while a positive MDM denotes hypermethylation in cases relative to controls. The rank is computed as the maximum (i.e., worst) of 3 ranks: (a) the mean difference in means across all sites in a region of the two groups being compared (MDM), (b) the mean of quotients in mean methylation, and (c) a combined *p*-value calculated from all site *p*-values in the region. Chr: chromosome.

**Table 3:** Summary of gene set enrichment analysis results.

Entity	Reactome Pathway Name	Size	ES	NES	*p-*Value	FDR q-Value
Genes	Glucuronidation	15	−0.84	−2.13	<0.001	<0.001
	Chondroitin sulfate dermatan sulfate metabolism	42	−0.57	−1.76	0.002	0.088
	Gap junction trafficking	25	−0.59	−1.65	0.002	0.228
	G β: γ signalling through PI3Kgamma	24	−0.59	−1.65	0.003	0.191
	Activation of kainate receptors upon glutamate binding	29	−0.55	−1.60	0.008	0.236
	Immunoregulatory interactions between a lymphoid and a non-lymphoid cell	58	0.31	1.49	<0.001	0.246
Promoters	Regulation of β cell development	27	−0.64	−1.92	<0.001	0.022
	Regulation of gene expression in β cells	17	−0.64	−1.75	0.002	0.200
	Immunoregulatory interactions between a lymphoid and a non-lymphoid cell	33	0.53	2.20	<0.001	0.007

Pathways with a false discovery rate (FDR) ≤0.25 are shown. Size, number of pathway genes available for analysis; ES, enrichment score for pathway; NES, normalized enrichment score for pathway.

**Table 4:** Enriched GO terms (*p* ≤ 0.005) among hypo- and hypermethylated regions.

ID	*p*-Value	Term	Region
Hypomethylated regions			
GO:0060337	8.00 × 10^−4^	type I interferon signaling pathway	genes
GO:0034340	9.00 × 10^−4^	response to type I interferon	genes
GO:0070458	2.10 × 10^−3^	cellular detoxification of nitrogen compound	genes
GO:0018916	2.80 × 10^−3^	nitrobenzene metabolic process	genes
GO:0060708	2.80 × 10^−3^	spongiotrophoblast differentiation	genes
GO:0032020	4.10 × 10^−3^	ISG15-protein conjugation	genes
Hypermethylated regions			
GO:0001837	1.00 × 10^−4^	epithelial to mesenchymal transition	genes
GO:0072079	1.00 × 10^−4^	nephron tubule formation	genes
GO:0048762	3.00 × 10^−4^	mesenchymal cell differentiation	genes
GO:0060980	1.00 × 10^−3^	cell migration involved in coronary vasculogenesis	genes
GO:0048729	1.70 × 10^−3^	tissue morphogenesis	genes
GO:0035295	1.70 × 10^−3^	tube development	genes
GO:0048864	1.80 × 10^−3^	stem cell development	genes
GO:0003218	1.90 × 10^−3^	cardiac left ventricle formation	genes
GO:0070172	1.90 × 10^−3^	positive regulation of tooth mineralization	genes
GO:0072272	1.90 × 10^−3^	proximal/distal pattern formation involved in metanephric nephron development	genes
GO:0072088	2.10 × 10^−3^	nephron epithelium morphogenesis	genes
GO:0061333	2.20 × 10^−3^	renal tubule morphogenesis	genes
GO:0060166	2.90 × 10^−3^	olfactory pit development	genes
GO:0060021	2.90 × 10^−3^	palate development	genes
GO:0060993	3.20 × 10^−3^	kidney morphogenesis	genes
GO:0072080	3.20 × 10^−3^	nephron tubule development	genes
GO:0045893	3.50 × 10^−3^	positive regulation of transcription, DNA-templated	genes
GO:0003166	3.90 × 10^−3^	bundle of His development	genes
GO:0072086	3.90 × 10^−3^	specification of loop of Henle identity	genes
GO:0072513	3.90 × 10^−3^	positive regulation of secondary heart field cardioblast proliferation	genes
GO:2000653	3.90 × 10^−3^	regulation of genetic imprinting	genes
GO:1902680	3.90 × 10^−3^	positive regulation of RNA biosynthetic process	genes
GO:0048598	4.20 × 10^−3^	embryonic morphogenesis	genes
GO:0051891	4.80 × 10^−3^	positive regulation of cardioblast differentiation	genes
GO:0072334	1.70 × 10^−3^	UDP-galactose transmembrane transport	promoters
GO:0035524	3.30 × 10^−3^	proline transmembrane transport	promoters
GO:0060166	3.30 × 10^−3^	olfactory pit development	promoters
GO:2000097	3.30 × 10^−3^	regulation of smooth muscle cell-matrix adhesion	promoters
GO:0001867	5.00 × 10^−3^	complement activation, lectin pathway	promoters
GO:0015820	5.00 × 10^−3^	leucine transport	promoters
GO:0019858	5.00 × 10^−3^	cytosine metabolic process	promoters
GO:0038110	5.00 × 10^−3^	interleukin-2-mediated signaling pathway	promoters

Enrichment of Biological Process (BP) Gene Ontology (GO) terms associated with the top-ranking 100 hypomethylated (top), and the top-ranking 100 hypermethylated (bottom) genes and promoters, as determined by a hypergeometric test implemented in RnBeads [29].

## Data Availability

The data presented in this study are openly available in the NCBI Gene Expression Omnibus (GEO) repository (accession code GSE150592). All the data are also available from the authors on request.

## Supplementary Materials

The following are available online at https://www.mdpi.com/2073-4425/12/2/129/s1: Table S1: Enrichment of DMC in different genomic locations. Figure S1. Manhattan plot of association of differential DNA methylation with SSc.
