# Supplementary material for: Differential DNA Methylation Landscape in Skin Fibroblasts from African Americans with Systemic Sclerosis
Source: Genes (Basel). 2021 Jan 20;12(2):129. doi: 10.3390/genes12020129 (PMC7909410; doi:10.3390/genes12020129)
Supplement: Supplementary file 1 [file genes-12-00129-s001.pdf]

Supplementary data

Table S1 Enrichment of DMC in different genomic locations

Region	Total CpGs	DMC	OR	95% CI	*p*-value
3' UTR	43386	17	1.29	0.80-2.07	0.3032
CpG Island	458155	80	0.57	0.46-0.72	<0.0001
Promoter	637936	64	0.33	0.26-0.42	<0.0001
TTS	80580	38	1.55	1.12-2.14	0.0077
Intron	504831	259	1.68	1.47-1.93	<0.0001
Exon	369552	115	1.02	0.84-1.24	0.8342
5' UTR	88242	5	0.19	0.08-0.45	<0.0001
Intergenic	343406	153	1.46	1.23-1.73	<0.0001
ncRNA	26039	8	1.01	0.50-2.02	0.9828
LINE	84771	26	1.01	0.68-1.48	0.9761
SINE	981925	359	1.20	1.07-1.35	0.0026

Genomic location of DMC that met an adjusted P value < 0.4. OR, 95% CI, and *p*-values were computed against the general distribution of the 3,870,251 CpGs of our dataset using GraphPad Prism. OR indicate enrichment or depletion of DMCs in each region.

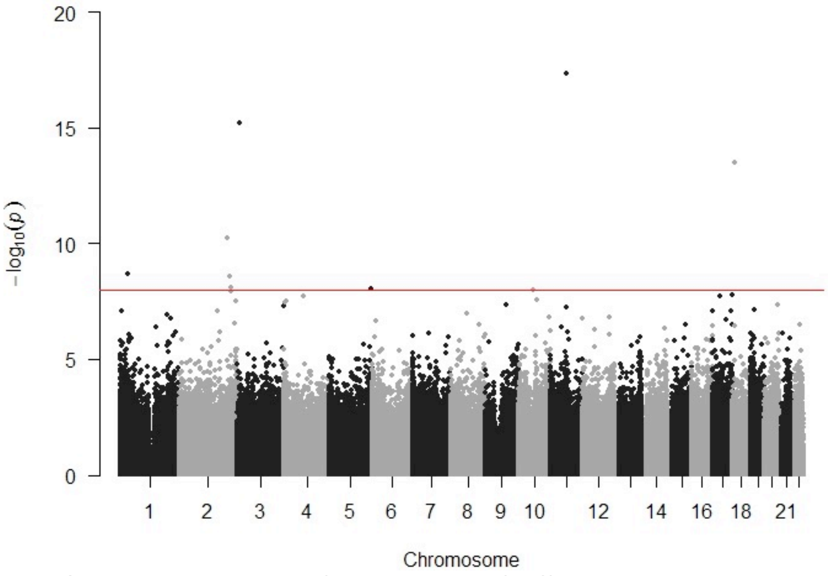

Fig. S1 Manhattan plot of association of differential DNA methylation with SSc. Each point represents a CpG (*n*=3,870,251) with the chromosomal position along the *x*-axis and the $-\log_{10}$(*P* value) on the *y*-axis. The red line corresponds to a Bonferroni corrected genome-wide significance level of 1.0×10^{-8}.
